# Venesection

**Published:** 1890-08-09

**Authors:** 


### VENESECTION.

The practice of bleeding from being of old regarded as a panacea, as not only relieving local congestion and general blood pressure or arterial tension, and allaying febrile and inflammatory processes, but in some mysterious manner removing every *materia peccans* from the system (though how such offending matter should be withdrawn from the larger volume of blood left behind was not explained) has latterly, except in the modest form of the occasional application of a few leeches, almost entirely fallen into disuse, not to say oblivion. Dr. Ferruccio, in four consecutive numbers of the *Gazzetta Degli Ospitali*, has given an able and learned review of the whole question from the several standpoints of history, pathology, and clinical practice; and arrives at the conclusion that, great as has been the abuse of blood-letting, there are cases in which it ought not to be neglected, maintaining its value in pulmonary œdema, suffocative catarrh, acute scarlatinal nephritis, in some cases of pneumonia, and even in a few of apoplexy in plethoric individuals, as well as in uræmic eclampsia, in which he has himself seen the best results.

Attributing its general disuse to the influence of such zealous opponents as Louis, Skoda, and Hughes Bennett, he appeals in favour of its occasional employment not only to his own experience but to such unimpeachable authorities as Niemeyer, Cantani, Bacelli, and Broadbent. Niemeyer, whose treatment was in general far from heroic, and always lucid and rational, recommended it in pneumonia when the patient was robust, the temperature over 40 deg. C. =104 deg. F., and the pulse above 120, and strong, and especially when the parts of the lung not involved in the pneumonic process were the seat of collateral œdema. Cantani, editing Niemeyer's great work, advises recourse to venesection in apolexy when the patient is robust, and the heart's action is still strong. Broadbent considers it very useful in acute nephritis, often causing the albumen to disappear from the urine, an effect which Ferruccio can confirm, and he agrees with Bacelli as to its indisputable value in uræmia, as he has often witnessed in the wards of the great clinical professor at Rome. He concludes his admirable essay with these suggestive and thoughtful words: "In the history of medicine it appears that phlebotomy has gone through many vicissitudes, passing from gross abuse to positive proscription. *Sit modus in rebus.* After so much controversy, the fact remains that venesection may be necessary in some contingencies. The question must be studied anew, without preconception or partisanship. In medicine, as in other sciences, questions again arise which were believed to have fallen into total oblivion."

